# Effects of Supplementation with Natural Antioxidants on Oocytes and Preimplantation Embryos

**DOI:** 10.3390/antiox9070612

**Published:** 2020-07-12

**Authors:** Maria Cristina Budani, Gian Mario Tiboni

**Affiliations:** 1 Dipartimento di Medicina e Scienze dell’Invecchiamento, Università “G. d’Annunzio” Chieti-Pescara, Via Dei Vestini 31, 66100 Chieti, Italy; maria.budani@unich.it; 2 Dipartimento di Scienze Mediche, Orali e Biotecnologiche, Università “G. d’Annunzio” Chieti-Pescara, Via Dei Vestini 31, 66100 Chieti, Italy

**Keywords:** infertility, in vitro fertilization, oxidative stress, natural antioxidants

## Abstract

For most infertile couples, in vitro fertilization (IVF) represents the only chance to conceive. Given the limited success of IVF procedures, novel approaches are continuously tested with the aim of improving IVF outcomes. Growing attention is devoted today to the potential benefit of natural antioxidants in the optimization of infertility treatments. This review summarizes current data in this context, focusing on both experimental studies on oocytes/embryos and clinical trials on antioxidants supplementation. Based on information gained from experimental studies, antioxidant supplementation may have beneficial effects on IVF outcomes in terms of quality and cryotolerance of in vitro produced embryos, together with positive effects on in vitro maturation oocytes and on early embryonic development. Unfortunately, from the clinical side, there is a paucity of evidence favoring the protective qualities of antioxidants. Among the antioxidants considered, coenzyme Q10 may be regarded as one of the most promising for its positive role in rescuing the oxidative stress-induced damages, but further data are needed. It is concluded that further trials are necessary to characterize the potential clinical value of antioxidants in IVF treatments.

## 1. Introduction

Infertility is the failure to establish a clinical pregnancy after 12 months of regular and unprotected sexual intercourse, and affects between 8–12% of reproductive-aged couples worldwide [1]. In vitro fertilization (IVF) represents an effective treatment for the couple who fail to conceive. Despite the percentage of successes, one of the most plausible causes of the failure of IVF procedures is the poor quality of gametes leading to aberrant embryonic development [2,3,4]. Among the mechanisms involved for the correct embryo development, the balance between reactive oxygen species (ROS) production and their detoxification becomes essential [5,6,7]. The oxidative stress (OS) condition occurs when the generation of ROS and other radical species (for example, reactive nitrogen species (NOS)) exceeds the scavenging capacity by antioxidants, due to excessive production of ROS and/or inadequate supplements intake of antioxidants [8] and/or inactivation of antioxidant enzymes. In vitro fertilization techniques, in particular, gametes collection, manipulation, and culture may generate reactive oxygen species (ROS) [9,10,11] with a possible role in impairing oocyte quality, sperm efficiency and, consequently, embryos development [12,13].

It is important to underline that oxidative stress exerts positive and negative effects on reproduction [12]. Adequate amounts of ROS play important roles in multiple physiological activities both in ovaries (from oocyte maturation to fertilization), and in the uterus [12]. The promotion of the development of follicles, from the primordial stage to antral follicles, [12] and the process of ovulation [14,15,16,17,18] require low levels of ROS. ROS and antioxidants are also connected to progesterone synthesis in the luteal phase [19]. In addition, physiological levels of ROS are essential in the regulation of the sperm function during the fertilization process [20]. ROS may exert physiological roles acting as second messengers, or may represent a source of cellular damage based on the level of their production. Among the physiologic functions, the mitochondrial activity, including the mitochondrial biogenesis and the mitochondria antioxidant capacities, demand a low level of oxidative stress [20,21]. Indeed, contrarily to the physiological roles, the excessive production of ROS negatively influences the quality of gametes. Mammalian spermatozoa are highly sensitive to significant ROS concentration [22], being the polyunsaturated fatty acids in their membrane highly susceptible to peroxidation. Excessive amount of ROS negatively influences oocyte maturation and oocyte fertilization [23,24,25,26,27,28,29], together with a role in decreasing sperm motility, sperm number, and sperm–oocyte fusion [20,30], with deleterious impact on embryo development. Considering that preimplantation embryos are particularly sensitive to their environment, which can impact their developmental potential [31,32], various approaches have been taken to improve in vitro development of embryos. Enzymatic and synthetic (dietary) antioxidants are the main defense factors against oxidative stress induced by free radicals [33]. Enzymatic antioxidants include superoxide dismutase (SOD), glutathione peroxidase (GPx), catalase (CAT), glutathione reductase (GSR), peroxiredoxins, and non-enzymatic antioxidants, known as natural dietary supplements, including vitamins and minerals [12,34,35]. Natural antioxidants, widely distributed in food (fruits, vegetables, cereals, mushrooms, beverages, flowers, spices, and traditional medicinal herbs), exhibit an extensive range of biological effects, such as anti-inflammatory, antibacterial, antiviral, anti-aging, and anticancer properties [34].

In this context, different studies tested natural non-enzymatic antioxidants supplementation for its potential influence on IVF outcomes. The focus of this review is to summarize research evidence on this matter. The results of in vitro and in vivo studies using animal models are summarized in Table 1. Clinical findings are summarized in Table 2. Data from experimental and clinical studies where antioxidants and vitamins were used in combination are reported in Table 3 and Table 4, respectively.

## 2. Agents Considered

### 2.1. Antioxidants

#### 2.1.1. Resveratrol

Resveratrol is a natural phytoalexin, a polyphenol synthesized by plants [85] and present in the skin of red grapes, red wine, and other botanical extracts [85]. An increasing number of studies have alluded to the potential benefit of resveratrol, including anti-cancer, anti-inflammatory, anti-senescence, and antioxidant properties [86]. Resveratrol influences the expression of a great number of enzymes including kinases, lipoxigenases, cycloxigenases, sirtuins, and transcription factors related to DNA synthesis, cell cycle, proliferation, stress responses, and apoptosis genes [36]. 

Concerning the role in IVF, the effects of resveratrol supplementation on the quality and cryotolerance of in vitro produced embryos [37,38,39], together with the positive effects of resveratrol on in vitro maturation oocytes and on early embryonic development [36,40,41,42,43,44], was assessed using experimental models. A preliminary dose-finding study, carried out with an in vitro study on bovine embryos, revealed that resveratrol up to 0.5 µM concentration is not affecting embryo quality. On the other hand, evident toxic effects were seen when concentrations higher than 5 µM were tested [37]. The dose-response study highlighted that significant decreases of both cleavage-stage embryos and blastocysts formation rates resulted from the addition of 5 µM resveratrol to culture medium. Failed blastocysts production was observed when 10 µM resveratrol was added to culture medium. This dose-dependent effect was attributed to the fact that while low levels of resveratrol display pro-proliferative and anti-apoptotic properties, high levels of resveratrol exert pro-apoptotic effects [38]. 

Bovine embryos cultured in vitro with 0.5 µM of resveratrol and then cryopreserved showed higher survival rates and hatching rates after warming, compared to the embryos cultured in absence of resveratrol [37]. In accordance with this study, Salzano et al. reported that treatment with 0.5 µM resveratrol improved the cryotolerance of in vitro bovine embryos [38]. The process of vitrification/warming was associated to an increased number of active mitochondria and increased mitochondrial superoxide production in bovine embryos [39]. The addition of 0.5 µM resveratrol to the in vitro culture medium or to vitrification/warming solutions induced an attenuation of the active mitochondria increase, but not an attenuation of mitochondrial superoxide production. On the contrary, the addition of resveratrol to both the in vitro culture medium and vitrification/warming solutions resulted in the attenuation for both the parameters (increase in active mitochondria and in mitochondrial superoxide production) [39]. This suggested a contribution of resveratrol in recovering from a low oxidative metabolism in bovine embryos [39]. Resveratrol has been also found helpful in supporting normal embryonic development [36,44]. The supplementation with 2 µM of resveratrol to in vitro maturated bovine oocytes improved the developmental potential of parthenogenically-active and IVF-produced porcine embryos [40]. In addition, the incubation of germinal vesicle-stage oocytes with resveratrol (1 µM) was associated with an increased cumulus cells expansion, polar body formation, and higher blastocysts rate, together with a higher number of cells in blastocysts [42]. Unfortunately, there is limited information concerning the effect of resveratrol supplementation on human IVF cycle outcome. One study, involving 102 women with regular resveratrol (200 mg/day) supplementation during IVF-ET cycles, associated resveratrol intake with a decrease in clinical pregnancy rate and an increase in the risk of miscarriage [61]. Embryo transfer cycles with poor prognostic factors were excluded from the study. Moreover, women with a history of resveratrol intake and no clinic revisits after embryo transfer and embryo transfer cycles using blastocysts were also excluded. The small number of patients included and the retrospective nature were the major limitations of the study. Bahramrezaie et al. conducted an interventional, triple-blind randomized clinical study involving 61 polycystic ovarian syndrome (PCOS) patients. All patients took resveratrol 800 mg/day (*n* = 30) or placebo (*n* = 31) for 40 days (from the beginning of their previous menstruation cycle until the oocyte retrieval day). The study suggested that resveratrol improved some outcomes of PCOS patients undergoing IVF (high-quality oocyte and high-quality embryo rates), probably through altering the serum levels of some sex hormones and the expression of VEGF and HIF1 genes in the angiogenesis pathway of granulosa cells [62]. In detail, the expression of both VEGF and HIF1 genes in granulosa cells was significantly lower in the resveratrol group compared to the placebo group [62].

#### 2.1.2. Melatonin

Melatonin is a pineal secretory product regulating circadian rhythms [87]. Several studies have documented its capacity in scavenging ROS [88], also within ovarian follicles [88]. Aged mouse oocytes cultured in vitro for 24 h showed an increased fertilization rate when supplemented with 10^−3^ M melatonin [45]. Moreover, melatonin restored the ability of sperm to bind to aged oocytes and reduced aging-induced ROS levels in post-ovulatory aged oocytes [45]. A positive effect of melatonin (10^−9^ M) on cleavage rates and in terms of blastocysts total cells number was seen in porcine embryos [46]. Melatonin improved mitochondrial function in mice oocytes matured in vitro, and protected oocytes matured in vitro from oxidative damages [47]. In detail, melatonin supplementation has a role in improving the activity of mitochondria. The mtDNA copy number, the degree of mitochondria granulated clustering, together with the mitochondrial membrane potential, resulted increased with melatonin supplementation in MII-stage oocytes [47]. Melatonin also manifested the ability of protecting embryos from the damaging effects of different stressors, such as heat [46,47,48] and H_2_O_2_ [46]. In an in vivo study, melatonin ameliorated the female reproductive performance in the mouse [49]. ICR mice, aged 7 weeks, were exposed to increasing concentrations of melatonin in drinking water (0, 3, 30, 300 µg/mL) for 21 days. A concentration of 30 µg/mL was associated to the higher number of antral follicles per area of ovarian structures, compared to control. Moreover, the IVF-derived hatched blastocyst rate in the group exposed to 30 µg/mL of melatonin was significantly higher in comparison to the control group, and the highest among the other melatonin-treated groups [49].

Much more controversial are the results provided by clinical studies. A recent meta-analysis failed to find a correlation between melatonin supplementation with the relevant IVF outcomes, including clinical pregnancy rate, number of oocytes retrieved, ovarian hyperstimulation syndrome (OHSS) risk, and miscarriage rate per clinical pregnancy rate [63]. A study [64] looked at the effect of oral melatonin administration of 3 mg/day during COS (for two weeks, ending on the day of hCG). The study compared the IVF outcome cycles of the same patient who completed two treatment cycles, with the first without supplementation, and the second cycle with melatonin [64]. Fertilization and good quality embryos rates were significantly higher in the group supplemented with melatonin with the fertilization rates ranging from 69.3% and 77.5% between the first cycle and second cycle. The rate of good quality embryos also increased in the second treatment cycle (48.0 versus 65.6%) [64]. In accordance to this study, two administrations of melatonin (3 mg/die and 6 mg/die) to patients with unexplained infertility (from the first appointment of COS until the day of ovum pick up procedure) resulted in a positive impact on IVF procedure [65]. In particular, irrespective of the two doses tested, the proportion of mature oocytes, fertilized oocytes, and the number of embryos transferred were higher in patients treated with melatonin compared to controls [65]. These results, however, have not been substantiated by others. A recent randomized placebo-controlled trial involving 160 patients (eligible for the study if they were undergoing their first cycle of IVF/ICSI and an antagonist cycle and if they were aged between 18 and 45, with a BMI between 18 and 35), failed to find any benefit after melatonin supplementation. Indeed, patients receiving melatonin at 2, 4, or 8 mg twice day from day 2 of their cycle until the night before oocyte retrieval did not manifest any benefit in terms of clinical pregnancy rate and oocytes and embryos parameters in comparison to the placebo group [66]. The levels of melatonin in the follicular fluid was the objective of the investigation of two studies supporting the idea that melatonin levels in follicular fluid may have a role as marker of ovarian reserve [89,90]. In particular, the presence of melatonin in follicular fluid was associated with the quantity and quality of oocytes [89,90], and with the antral follicle count (AFC), serum anti-Müllerian hormone (AMH) level, serum estradiol level, and number of total embryos and blastocysts obtained [90]. The molecular basis whereby melatonin exerts its effect on oocytes and embryos include a direct antioxidant effect (non-receptor mediated). The indirect actions are mediated via cell membrane receptors (MT1, MT2) and nuclear receptor (RORα), and culminate in the regulation of the expression of genes connected to oocyte maturation and embryonic development [66]. 

#### 2.1.3. Coenzime Q10

Coenzime Q10 (CoQ10) is an electron carrier in mitochondria also acting as scavenger against reactive oxygen species [91]. In vitro and in vivo studies investigated the role of CoQ10 supplementation [50,51,52,53], mostly, in the field of maternal aging and obesity. The positive role of CoQ10 in ameliorating the quality of postovulatory aged oocytes, the competency, together with the fertilization capacity of aged gametes, was documented in vitro [50]. Considering that mitochondrial dysfunction has been implicated in oocyte aging, Ben-Meir and colleagues assessed, in an aged animal model, whether CoQ10 treatment could improve fertility and mitochondrial performance in mouse oocytes [51]. The study suggested that CoQ10 supplementation delayed the depletion of ovarian reserve, restored the oocyte mitochondrial gene expression, and improved the mitochondrial activity [51]. Specifically, the CoQ10-treated aged animals showed an increased expression of Sdha and Nduf3, Sod1 (mitochondrial ROS scavenger), as well as Smarca2 (ATP-dependent chromatin regulator) that were all significantly decreased in the oocytes of aged animals untreated with CoQ10 [51]. 

The capacity of CoQ10 in ameliorating the quality of oocytes recovered from obese mice was also shown by an in vivo study [52]. Normal and obese mice were assigned to receive a subcutaneous dose of CoQ10 (22 mg/kg, 3 times/week). Some of the obesity-induced effects on oocytes were prevented by CoQ10 supplementation, with mitochondrial distribution, spindle formation, and chromosome alignment improvement [52].

With respect to human studies, the levels of CoQ10 in follicular fluid were related to embryo grades and pregnancy rates, with high-quality embryos and better pregnancy rate associated with higher CoQ10 levels [92]. Concerning the maternal aging, a clinical investigation strengthened the beneficial properties of CoQ10 demonstrated in animal studies on this matter. Oral CoQ10 administration (200 mg/day in two daily administration, for 30 days) improved the follicular fluid oxidative metabolism and the oocyte quality in over 35-year-old-women [67]. A controlled randomized trial with an oral CoQ10 supplementation of 600 mg for two months and for up to three cycles (if pregnancy did not occur) resulted in a lower rate of aneuploidy in post-meiotic oocytes retrieved from aged women [68], but no significant differences in IVF outcomes were detected between the CoQ10 and placebo groups [68]. 

The beneficial effects of CoQ10 supplementation were also observed in young women [69]. In young women with low ovarian reserve and supplemented with CoQ10 (200 mg, three times a day for 60 days), an increased number of retrieved oocytes, fertilization rate, and high-quality embryos transferred were noted. The clinical pregnancy and live birth rates per embryo transfer and per one complete stimulation cycle tended to be higher in the CoQ10 group, albeit without statistical significance [69]. 

It has been suggested that CoQ10 counteracts physiological ovarian ageing by restoring mitochondrial function. CoQ10 functions as an electron carrier in the mitochondrial respiratory chain and has a key role in oxidative phosphorylation to produce adenosine triphosphate (ATP). In addition, CoQ10 exerts a crucial role as antioxidant by inhibiting lipid peroxidation and DNA oxidation, strengthening the endogenous antioxidant system [69].

Overall, current data support the beneficial effects of CoQ10 in restoring the damages inducted by maternal aging and obesity, although the effectiveness of this compound in the context of IVF outcomes needs to be confirmed by further studies.

#### 2.1.4. Antioxidants in Combination: Acetyl-l-Carnitine + N-Acetyl-Cysteine + α-Lipoic-Acid and β-Mercaptoethanol + Cysteamine

Experimental in vitro studies considered the effectiveness of antioxidants when given in combination. Truong and colleagues (2016, 2017) looked at the effects of the addition of a combination of triple antioxidants (10 µM acetyl-l-Carnitine, 10 µM *N*-acetyl-Cysteine- 5µM α-lipoic-acid) in the culture medium of mouse pronucleate oocytes and preimplantation embryos [78,79]. L-carnitine reduces ROS levels with its antioxidant actions and acts through the regulation and transport of long chain fatty acids into mitochondria for β-oxidation and ATP production. As well, α-lipoic acid regulates mitochondrial function and ATP production and stimulates the expression of antioxidant genes involved in defense mechanisms against oxidative stress. *N*-acetyl cysteine acts through the up-regulation of glutathione (GSH) synthesis, protecting from oxidative stress [78].

There was a beneficial effect of these combined antioxidants on embryo development [78], and a positive correlation between the presence of these antioxidants and increased blastocyst cells number [79]. The evidences about the efficacy of these compounds, administered alone in culture medium, in increasing the oocytes fertilization rate [93,94] and in improving embryo development [95,96,97,98,99] has also been highlighted in experimental studies [93,94,95,96,97,98,99].

β-mercaptoethanol and cysteamine are thiol compounds that stimulate GSH synthesis. GSH plays central roles in oocyte maturation and in protecting them from damages reactive oxygen species-induced [80]. In a mouse model, in vitro maturation (IVM) of immature oocytes and IVF outcome resulted favored by the concomitant supplementation of 100 µM β-mercaptoethanol and cysteamine in culture media [80]. These findings are in line with the results obtained using a bovine in vitro system [81]. A dose-dependent influence of β-mercaptoethanol and cysteamine, administered alone in culture medium on oocyte maturation and embryo development after IVF has also been highlighted [100,101,102].

### 2.2. Vitamins

#### 2.2.1. β-Carotene

Carotenoids have gained attention in the reproductive field because they function as potent antioxidants by scavenging ROS [103]. The role of β-carotene in promoting the citoplasmatic in vitro maturation of bovine oocytes has been highlighted [104]. Furthermore, the retinoic acid (RA), a central metabolite of vitamin A, has a role in promoting the cytoplasmic maturation of bovine oocytes by controlling the gene expression of gonadotropin receptors, cyclooxygenase-2 and nitric oxide synthase (NOS) in cumulus-granulosa cells [104]. A recent study showed that the inhibition of oocyte development/maturation and parthenogenetic activation oxidative stress-induced by ROS could be rescued by β-carotene in vitro. The mechanisms whereby β-carotene rescues the negative effects of ROS on oocyte development and maturation is not limited to the mitigation of ROS generation and cell apoptosis index. Indeed, β-carotene played a role in regulating the actin expression, together with the formation of cortical granule-free domain (CGFD) and the homogeneous distribution of mitochondria [105].

There is a paucity of data in literature concerning the effects of β-carotene on human fertility and regarding its potential effects on IVF programs outcome. A study determined the concentrations of carotenoids, retinol and α-tocopherol in follicular fluid and plasma in women undergoing IVF programs [106], and showed that differences between plasma and follicular fluid were greater for β-carotene and lycopene (<20% of plasma concentration) [106]. Moreover, an exogenous stress, such as cigarette smoke, influenced the level of β-carotene in women undergoing IVF treatments, with smokers having significantly lower levels of follicular fluid β-carotene in comparison to non-smokers [107]. In accordance, the mean β-carotene levels in follicular fluid and plasma of smokers were found to be lower compared to the levels in the non-smokers group [108]. It remains to be elucidated the potential consequences of reduced β-carotene levels in follicular fluid microenvironment on oocyte maturation process. The possible effects of β-carotene deficiency on IVF outcomes, and the role of β-carotene supplementation appears to be points deserving clarification.

#### 2.2.2. B-Vitamins

Vitamins B are water-soluble compounds with antioxidant properties [109]. The role of B-vitamins in the field of fertility has been the subject of in vitro and in vivo studies [54,55]. The effects of vitamin B_9_ (folic acid) were tested in vitro by exposing mouse oocytes treated with hypoxantine (inhibitor of mouse oocyte maturation) to 500 µM of folic acid. The deranging effects of hypoxantine were reversed when the culture medium was added with folic acid, which restored the oocytes spindle configuration and the distribution of cortical granules [54].

Severe B_1_ deficiency diet was linked to alterations in the meiotic maturation of oocytes in a mouse model [55]. While the frequency of abnormal oocytes in ICR mice fed with a vitamin B_1_-free diet did not differ from that of the control groups after 13 days of diet, prolonging the vitamin B_1_-free diet up to 20 days lead to an increase of abnormal oocytes in the test mice than in the control group. Aberrant eggs presented spindle defects and growth arrest at the germinal vesicle (GV) stage. After feeding mice with the nutritionally complete diet, oocyte abnormalities were partially reverted, suggesting that vitamin B_1_ deficiency acted by inhibiting oocytes meiotic maturation trough a reversible process [55].

Concerning the effects of B-vitamins on human female fertility, particular attention was devoted to folate (vitamin B_9_) and vitamin B_12_. The consequences of folate deficiency and defects in folate and homocysteine metabolisms on female fertility have been recently examined [110], with findings highlighting an increased risk of ovulatory deficiency, longer time to pregnancy, and neural tube defects for folate-deficient women [110]. Noteworthy, a recent study revealed that serum and red blood cells folates were largely inadequate among women attending an IVF program [111]. Vitamin B_12_ levels were largely insufficient in women attending ART. The use of dietary folic acid supplementation had no significant effect on vitamin B_12_ serum levels [111].

Further studies demonstrating the importance of proper folate levels for women attending IVF treatments is the notion that higher levels of serum and red blood cell folates confers a higher chance of becoming pregnant [112]. A recent study investigated the serum folate, homocysteine, and vitamin B_12_ levels in 77 women entering an in vitro fertilization program, highlighting that only a portion of patients presented proper folate levels [113]. Two prospective studies involving women undergoing infertility treatments showed that women with a high level of folate had better IVF outcomes [70,71]. The diet assessment in these studies was carried out by a food frequency questionnaire, assessing the folate intake of women recruited in the analysis. The folate status was assessed in red blood cells and plasma [70] and in serum [71]. For both the studies, women were eligible for the analysis if they had completed a food-frequency questionnaire and had subsequently completed at least one ART cycle. Moreover, they resulted eligible if they had not planned the use of donor gametes at enrolment [70,71]. In the first study, including 232 women, the total folate intake was positively associated with implantation, clinical pregnancy, and live birth rates per initiated cycle, and the pre-treatment with supplemental 800 μg/day of folic acid resulted in a higher probability of live birth. The live birth rate among women consuming 800 μg/day folic acid was 20% higher compared to women consuming 400 μg/day folic acid [70]. In keeping with these findings, a cohort study involving 100 women reported that the probability of live birth is 2 times higher in patients with a serum folate > 26.3 ng/mL compared to women with serum folate < 16.6 ng/mL and in patients with serum vitamin B_12_ > 701 pg/mL compared to those with serum vitamin B_12_ < 439 pg/mL [71]. In contrast with previous findings, two trials conducted by Murto et al. failed to associate folic acid intake or good folate plasma status, together with MTHFR gene variations, to a better pregnancy outcome following infertility treatment [72,73]. In accordance with Murto et al. [72,73] data, a prospective cohort study of 602 women undergoing infertility treatment, did not find association between folate and vitamin B_12_ levels and likelihood of a successful pregnancy [74].

Understanding the potential contribution of folates and vitamin B_12_ supplementation to the success in infertility treatments remains a crucial question, reinforcing the necessity for additional research.

#### 2.2.3. Vitamin C

Vitamin C (l-ascorbic acid) is a simple-low-molecular weight carbohydrate-like molecule that plays important roles in various enzymatic processes [109]. Ascorbic acid is known for its powerful antioxidant properties since it acts in reducing or scavenging of free radicals and ROS [114]. In vitro studies using animal models tested the efficacy of ascorbic acid in improving survival rate of porcine cryopreserved embryos. Results were compatible with the conclusion that supplementing culture and vitrification/warming media with L-ascorbic acid can improve the quality and the survival rates of porcine embryos after warming [56,57,58]. On the other hand, Nohalez et al. did not observe substantial effects of ascorbic acid supplementation (50 μg/mL) in any of the maturation, fertilization, or embryo development parameters, when added in in vitro fertilization and embryo culture media [58].

Several clinical trials evaluated the role of vitamin C supplementation during COS in IVF programs, offering controversial results. In a prospective randomized double-blind study conducted on 619 patients supplemented with three doses of vitamin C (1-5-10 gr) during the luteal phase of the in vitro fertilization-embryo transfer (IVF-ET) program (starting on the day of follicle aspiration for 14 days), no positive influence of ascorbic acid administration on clinical pregnancy and implantation rates was found [75]. Selection criteria were patients undergoing the first IVF cycle with age younger than 40 years. Cases with tubal, idiopathic, and male infertility were included in the study. On the contrary, patients suffering from renal or gastrointestinal disease and with repeated IVF cycles were excluded [75]. The notion that vitamin C is not providing benefits to IVF patients is also supported by the results of a randomized controlled study involving 280 patients with endometriosis (160 women supplemented with vitamin C and 120 not treated) and 150 patients without endometriosis used as the control group. Ascorbic acid was given (in a standard dose of 1000 mg/day) from two months before IVF-ET up two weeks after embryo transfer. There were no significant differences in the fertilization rate, implantation rate, or clinical pregnancy rate among the study groups [76]. Opposite results were reported by a prospective study involving 76 women (38 of them smokers and 38 non-smokers) and showing that vitamin C supplementation (500 mg) during the period of hormonal stimulation is associated with higher pregnancy rate, especially in non-smoker patients supplemented with vitamin C [77].

#### 2.2.4. Vitamin D

Vitamin D is a fat-soluble vitamin that regulates several pathways by binding the Vitamin D receptor [115]. Concerning the female reproductive system, ovaries, endometrium, and fallopian tube epithelial cells expressed vitamin D receptor [115]. Several studies supportive for a better chance of pregnancy in women replete for serum/follicular fluid vitamin D levels have been published. A meta-analysis pooling data from nine studies evaluating the association between vitamin D levels and the clinical outcomes after IVF/ICSI showed a trend toward lower clinical pregnancy and ongoing pregnancy rates in women with deficient levels of vitamin D [115]. The meta-analysis included both retrospective and prospective trials. Vitamin D levels were mainly evaluated in the women serum, and only in one study it was also assessed in the follicular fluid. Another meta-analysis conducted by Lv et al. reached the conclusion that deficient vitamin D levels were associated with a lower live birth rate in IVF women [116]. This metanalysis included five studies, with different design (both retrospective and prospective) and different source of vitamin D samples. Again, women replete in vitamin D showed more probability of clinical pregnancy and live birth compared to women with deficient or insufficient vitamin D status [117]. No association was found between miscarriage and vitamin D serum concentrations [117]. Overall, these data are encouraging vitamin D supplementation in deficient women undergoing infertility treatments. Nevertheless, further studies are needed to strengthen this idea, considering the heterogeneous scientific nature of the currently available reports. An advice to be selective in deciding for vitamin D supplementation was recently given [118,119], in view of the negative correlation between follicular fluid vitamin D levels and the quality of embryos [117], together with the lack of statistical support for lower pregnancy rates in vitamin D-deficient women in comparison with vitamin D-sufficient patients [118]. In this perspective, a protocol for performing randomized clinical controlled trial has been recently published [120].

#### 2.2.5. Vitamin E

Vitamin E is the predominant lipid-soluble antioxidant in animal cells, present also in the ovary and follicular fluid [121,122]. The possible link between vitamin E and improved embryo development was suggested by data derived by animal studies [59,60]. A complex study evaluated whether embriotoxicity (indicated by a reduced blastocyst development rate) could be reversed by adding antioxidants (vitamin E and vitamin C) for 3 and 6 h to the culture media supplemented with a possible source of ROS (12-phorbol 13 myristate acetate (PMA)-activated leukocyte) [59]. The blastocyst development rate increased after vitamin E supplementation (400 µM) at 6 h but this positive effect was not effective as that showed with the vitamin C supplementation (50 µM, 6 h) [59]. The role of vitamin E in enhancing embryonic development was noted in vitro in a study conducted on bovine model, where the embryo culture media supplemented with 100 mM vitamin E supported the development of more embryos to early and expanded blastocysts [60].

A study carried out in women undergoing IVF showed that, vitamin E present in the follicular fluid or serum, is related to higher oocyte maturation and higher quality embryos rates [123]. In particular, 0.35/1 mg/dL and 1.5/2 mg/dL are the levels of vitamin E in follicular fluid that allowed achieving the highest percentage of MII oocytes [123]. Similarly, 10/15 mg/dL serum level of vitamin E was related to the highest percentage of higher quality of embryos obtained [123]. A recent randomized controlled study including 105 patients showed that the concomitant administration of two vitamins (Vitamin E: 400 mg/day and vitamin D_3_:50000 IU/one in two weeks for 8 weeks) to PCOS women was associated to an increment of pregnancy, clinical pregnancy, and implantation rates in comparison to controls [82]. The limited clinical data about the effects of vitamin E to women undergoing IVF treatments call the necessity for future studies.

#### 2.2.6. Antioxidants and Multivitamins in Combination

Different studies have been published about the effects of antioxidants and multivitamins in combination to patients attending ART [82,83,84]. We recapitulated information of these studies in Table 4. Contrarily to the analysis conducted by Youssef et al. [84] evidencing no changes between the study groups as regarding the number of mature (MII) oocytes and clinical pregnancy rate, the data highlighted that the combination of different antioxidants and vitamins ameliorate the IVF treatments of infertile couples in terms of pregnancy rate [82,83]. However, the heterogeneity among the studies, including the different study design and the different formulations tested, prevent delineating robust evidence for the clinical use of micronutrients in combination during IVF treatments.

## 3. Conclusions

This manuscript reviewed the literature concerning the effects of natural antioxidants supplementation on IVF outcomes, considering both experimental studies and clinical trials.

In general, experimental studies using animal models have expressed support for a beneficial effect of antioxidant on the outcome of IVF techniques in terms of quality and cryotolerance of in vitro produced embryos, together with positive effects on in vitro maturation oocytes and on early embryonic development. Unfortunately, the protective qualities displayed by antioxidants with experimental models were supported only in part by human studies.

With respect to resveratrol, while in vitro studies suggested a helpful role [36,37,38,39,40,41,42,43], only few clinical studies found an improvement of IVF outcome after resveratrol supplementation [61,62]. Melatonin and vitamin C showed favorable effects in vitro, but clinical studies delivered controversial results [64,65,66,75,76,77]. With regard to vitamin C supplementation, the heterogeneity of the populations analyzed among the studies [75,76,77], together with the different study designs, create difficulties in outlining the effectiveness of vitamin C intake during IVF programs. β-carotene resulted helpful in in vitro studies in promoting the cytoplasmic maturation of oocytes [105,106], but there is a lack of clinical data on female fertility and, as supplementation, on IVF outcomes. Folates and vitamin B_12_ are the most studied B-vitamins in the human field in the context of IVF procedures [70,71,72,73,74], but sufficient evidence to support clinicians in making a decision is still lacking. The need to expand data also applies to vitamin D supplementation. A protocol for setting randomized clinical controlled trials has been recently published, in order to standardize the methodology of vitamin D supplementation during COS in IVF programs [120]. The CoQ10 supplementation showed positive effects both in young women with low ovarian reserve [69] and in aged women [67,68]. The properties of CoQ10 in restoring mitochondrial activity and to act as an antioxidant by inhibiting lipid peroxidation and DNA oxidation make this compound encouraging in the protection from oxidative stress-induced damages. The promising clinical data provide the groundwork for subsequent studies on CoQ10 supplementation.

Evidence in favor of antioxidant supplementation for IVF is accumulating in recent literature.

Experimental studies allow us to delineate the effects of antioxidants use in vitro or in vivo, and in particular, the molecular effects of these compounds on gametes and embryos. These positive results unfortunately are not in line with the conclusions offered by clinical trials. Differences among the trials in terms of study design, inclusion criteria, and statistical power make comparison difficult. Studies with less methodological heterogeneity along with larger human trials are necessary to define the role of natural antioxidants, alone or in combination, as supplementation among women undergoing infertility treatments. Another important aspect that should deserve more consideration relates to the potential protective role of natural antioxidants against endocrine disruptors-induced oxidative stress [124,125].

## Figures and Tables

**Table 1:** In vitro and in vivo studies about antioxidants and vitamins in in vitro fertilization.

Component	Authors	Animal Model	Study Design	Dose	Results
	Lee et al., 2010 [36]	Bovine	In vitro	0.5 µM	-Resveratrol increased the percentage of parthenogenically-activated and IVF-produced embryos reaching the blastocyst stage and the total cells number of blastocysts (↑)
	Abdel-Wahab et al., 2012 [37]	Bovine	In vitro	0.5 µM	-Resveratrol increased survival and hatching rates of embryos cryopreserved (↑)
	Salzano et al., 2014 [38]	Bovine	In vitro	0.5 µM	-Resveratrol increased development and hatching rates of embryos cryopreserved (↑)
	Gaviria et al., 2019 [39]	Bovine	In vitro	0.5 µM	-Resveratrol attenuated the increasing in active mitochondria in embryos cryopreserved (↑)
	Kwak et al., 2012 [40]	Porcine	In vitro	0.1, 0.5, 2.0, 10.0 µM	-Lower levels of intracellular ROS in oocytes matured in vitro (2.0 µM) (↑) -Higher blastocyst formation rates and total cells number after parthenogenic activation and IVF (2.0 µM) (↑) -Lower expression of apoptosis-related genes in COC treated with 2.0 µM resveratrol (↑)
	Liu et al., 2013 [41]	Mouse (C57BL6)	In vivo (aged 2–3 months) In vitro	Resveratrol added to drinking water at 30 mg/L for 6 or 12 months (in vivo) 0.1, 0.5, 1.0 µM (in vitro)	-Aged mice that received resveratrol delivered pups albeit with a reduced litter size (↑) -Increased number of primary and growing follicles (6 months of resveratrol intake) and prevention of telomere shortening together with an increase in telomerase activity (12 months) (↑) -Higher frequency of normal oocytes than age-matched untreated control (6 months of treatment) (↑) -Increased rate of development to blastocysts and increased total cells number in blastocysts (0.1 µM resveratrol in culture medium) (↑)
	Wang et al., 2014 [42]	Bovine	In vitro	0.1, 0.5, 1.0 µM	-Improved cumulus expansion, polar body formation, hatched blastocyst rate and mean number of cells in blastocysts (1.0 µM) (↑) -Resveratrol induced progesterone secretion and had antioxidants effects (↑)
	Li et al., 2018 [43]	Bovine	In vitro	0, 10^−3^, 10^−4^, 10^−5^, 10^−6^ M	-Resveratrol decreased ROS, phosphatidylserine externalization and malonialdehyde and protected mitochondrial function in sperm and acrosome integrity (10^−4^ M) (↑) -Increased blastocysts number and quality following IVF (10^−4^ M) (↑)
	Piras et al., 2020 [44]	Cat	In vitro	5 µM	-The rate of blastocyst formation, after IVM of oocytes incubated with resveratrol, was higher than that of oocytes matured without resveratrol (↑)
**Melatonin**	Dai et al., 2017 [45]	Mouse (ICR)	In vitro	10^−9^, 10^−7^, 10^−5^,10^−3^ M	-Increased fertilization potential and elevated sperm binding ability in post-ovulatory aged oocytes (10^−3^ M) (↑) -Decreased ROS and early apoptosis with melatonin supplementation (↑)
	R. Osorio et al., 2007 [46]	Porcine	In vitro	10^−12^, 10^−9^, 10^−6^,10^−3^ M	-Positive effects on cleavage rates and blastocysts cells number (10^−9^ M) (↑) -Decreased cleavage rates (10^−3^ M) (↓) -Melatonin (10^−9^ M) protection of embryos against heat stress (40 °C for three hours) (↑)
	He et al., 2016 [47]	Mouse (CD1)	In vitro	10^−5^, 10^−7^, 10^−9^ M	-Melatonin improved mitochondrial function (10^−5^ M and 10^−7^ M) together with mitochondrial distribution and ATP production in oocytes (10^−7^ M) (↑) -Role in reducing ROS formation (10^−7^ M) and in enhancing meiotic spindle assembly (10^−7^ M) (↑) -Melatonin improved IVF embryo development: higher blastocysts rate with 10^−5^ and 10^−7^ M melatonin and higher number of blastocysts cells with 10^−7^ M melatonin supplementation (↑)
	Serrano et al., 2013 [48]	Bovine	In vitro	10^−12^, 10^−9^, 10^−4^; 10^−3^ M	-10^−4^ M melatonin alleviated bovine oocytes from the harmful effects of heat stress (↑)
	Zhao et al., 2018 [49]	Mice (ICR)	In vivo (aged 7 weeks)	Melatonin added to drinking water at 0,3, 30, and 300 µg/mL for 21 days	-Litter size increased (3 µg/mL) (↑) -Higher antral follicles count and hatched blastocysts rate in 30 µg/mL group than control group (↑)
**CoenzymeQ10**	Zhang et al., 2019 [50]	Mice (ICR)	In vitro	25, 50, or 100 μM	-Role of CoQ10 supplementation in preventing aging damages (↑) -Number of sperm binding to the zona pellucida significantly restored in the CoQ10 supplemented group (↑) -Localization of Juno on the membrane in aged oocytes rescued with CoQ10 supplement (↑) -Levels of superoxide and DNA damage reduced with CoQ10 administration (↑)
	Ben-Meir et al., 2015 [51]	Mouse (ICR)	In vivo (For aging experiments, only retired breeders were used. Young controls were virgin females aged 7–8 weeks old)	Subcutaneous doses of ALA (33 mg/kg), resveratrol (10 mg/kg), CoQ10 (22 mg/kg), or placebo (sesame oil) three times a week for a period of at least 12 weeks. For experiments in the Pdss2 model, mothers during pregnancy and their offspring after weaning received CoQ10 in drinking water (0.4 mg/mL).	-Age-related decline in oocyte quality and quantity reversed by the administration of CoQ10 (↑) -Prevention of premature ovarian failure in the oocyte-specific Pdss2-deficient animals by maternal dietary administration of CoQ10 (↑)
	Boots et al., 2016 [52]	Mouse (C57BL6)	In vivo (aged 4 weeks)	22 mg/kg CoQ10 3 times/week dissolved in sesame oil, subcutaneously.	-Reduced levels of intracellular ROS in oocytes of mice treated with normal diet but not in those in high fat-high glucose diet -Higher percentage of normal spindles and chromosome alignment in oocytes of mice supplemented with CoQ10 (↑) -No differences in the number of mature oocytes, fertilization rate, blastocyst formation rates, implantation rates, resorptions rates, or litter size between obese mice receiving CoQ10 or vehicle (→)
	Maside et al., 2019 [53]	Porcine	In vitro	10, 25, 50, and 100 μM	-No effects on the percentage of MII oocytes, fertilization, and on the parameters of subsequent embryonic development (10–50 μM CoQ10 to the IVM medium) (→) -The highest concentration of CoQ10 (100 μM) in the maturation medium negatively affected blastocyst rates (↓)
**B-Vitamins**	Huang et al., 2013 [54]	Mouse (Kumming), Xenopus	In vitro	500 μM	-The deleterious effects of hypoxantine counteracted by folic acid (500 μM) (↑) -In folic acid-treated Xenopus eggs, extracellular signal-regulated kinase 1 was phosphorylated, cyclin B2 and Mos up-regulated and the frequency of GVBD accelerated (↑)
	Tsuji et al., 2017 [55]	Mouse (ICR)	In vivo (aged 5–6 weeks)	Female mice were fed a 20% casein diet (control group) or a vitamin B1–free diet (test group)	-Frequency of abnormal oocyte was increased by vitamin B_1_ deficiency when deficiency was accompanied by body weight loss -The frequency of abnormal oocytes decreased by refeeding of a vitamin B_1_–containing diet (↑)
**Vitamin C**	C. Martin et al., 2014 [56]	Porcine	In vitro	50 µM β-mercaptoethanol or 100 µM l-ascorbic acid	-ROS levels and survival rates after vitrification–warming significantly improved in embryos cultured with ascorbic acid (↑) -l-ascorbic acid into vitrification–warming media enhanced embryo survival and embryo quality after warming (↑)
	C. Martin et al., 2015 [57]	Porcine	In vitro	100 µM	-l-ascorbic-acid enhanced survival rates of blastocysts and reduced peroxide levels (↑) -No significant differences in total cells number, DNA fragmentation, and BAX, BCL2L1 and POU5F1 expression levels in vitrified blastocysts among experimental groups (→) -Vitrification procedures increased HSPA1A transcript abundance, but this increase was lowered with l-ascorbic acid supplementation (↑)
	Nohalez et al., 2018 [58]	Porcine	In vitro	50 µM	-No significant effects of l-ascorbic acid in any of the maturation, fertilization, or embryo development parameters assessed (→) -Blastocyst survival rate after vitrification increased with l-ascorbic acid addition to vitrification/warming media (↑) -Blastocysts intracellular ROS decreased with addition of l-ascorbic acid to vitrification/warming media but did not affect GSH (↑)
**Vitamin E**	Wang et al., 2002 [59]	Mouse	In vitro	Vitamin C (0 to 400 µM), and vitamin E (0 to 800 µM) (3–6 h)	-Increased blastocyst development rate co-incubating embryos with vitamin C (50 µM–3 h) and PMA-activated supernatant -Increased blastocyst development rate with vitamin E supplementation (400 µM) at 6 h (↑)
	Olson et al., 2000 [60]	Bovine	In vitro	100 mM vitamin E; 100 mM vitamin E + 100 mM vitamin C 100 mM vitamin E + 100 mM vitamin C + 3 mM EDTA	-More zygotes developed to expanded blastocysts (culture medium contained 100 mM vitamin E) (↑) -Combined vitamins E and C resulted in lower development to early, expanded, and hatched blastocysts than with vitamin E alone, as was the mean number of cells per blastocyst (→) -Addition of EDTA (3 mM) failed to improve development over that in culture with vitamin E + vitamin C (→) -Larger surface area of embryos cultured with vitamin E (↑)

**Table 2:** Human clinical studies about antioxidants and vitamins during controlled ovarian stimulation (COS) for in-vitro fertilization (IVF) programs.

Component	Authors	Study Design	Patients Enrolled	Dose Supplemented	Results
**Resveratrol**	Ochiai et al., 2019 [61]	Retrospective, cross-sectional	7277 embryo transfer cycles: -7023 cycles in 2958 women without resveratrol supplementation -204 cycles in 102 women with regular resveratrol supplementation	200 mg/day during IVF-embryo transfer cycles	-Resveratrol supplementation associated with a decrease in clinical pregnancy rate and increased risk of miscarriage (↓)
	Bahramrezaie et al., 2019 [62]	Triple-blind randomized controlled trial	61 PCOS patients: -31 supplemented with placebo (controls) -30 supplemented with resveratrol	800 mg/day for 40 days (from the beginning of their previous menstruation cycle until the oocyte retrieval day)	-The number of mature oocytes, cleavage rate, fertilization rate, and fertility rate were not significantly different between the two groups (→) -The high-quality oocyte rate and high-quality embryo rate were higher in the resveratrol group (↑)
**Melatonin**	Seko et al., 2014 [63]	Systematic review and meta-analysis of five randomized controlled trial	680 women undergoing ART from 5 studies were included with: -344 women not supplemented with melatonin -336 women supplemented with melatonin	Two studies compared melatonin supplementation (3 mg/day) during COS. The other three studies compared supplementation with melatonin + standard treatment (folic acid + myoinositol) versus standard treatment (folic acid + myoinositol): two of these used the following doses: myoinositol 4 g/day, folic acid 400 mg/day, and melatonin 3 mg/day. The other study did not report the doses used.	The estimates were imprecise for distinguishing between no effect and benefit considering clinical pregnancy, the number of oocytes retrieved, and for distinguishing among harm and no effects, considering miscarriage and risk of OHSS (→)
	Nishihara et al., 2014 [64]	Retrospective	Study designed for two consecutive IVF cycles. 194 cycles in 97 patients: -78 cycles (ICSI) and 19 (IVF) with no melatonin supplementation -83 cycles (ICSI) and 14 (IVF) with melatonin supplementation.	3 mg of melatonin orally, once a day, for at least 2 weeks, ending on the day of hCG injection at the second cycle.	-No significant differences in maturation rates, blastocyst rates, and good quality blastocysts rate between the first and second cycle (→) -The fertilization rate higher in the second cycle than that in the first cycle with the fertilization rate dramatically increased after melatonin treatment. The rate of good quality embryos also increased (↑)
	Espino et al., 2019 [65]	Randomized pilot study	40 women involved: -10 women no supplemented with melatonin; -10 women supplemented with a daily dose of 3 mg of melatonin; -women supplemented with a daily dose of 6 mg of melatonin.	Daily dose of 3 or 6 mg of melatonin. Melatonin was taken one hour before going to sleep for a period spanning from the first appointment to control ovarian stimulation until the day of follicular puncture, i.e., for 40 days	-Irrespective of the two doses tested, melatonin supplementation ameliorated intrafollicular oxidative balance and oocyte quality in patients with unexplained infertility, with increased rate of pregnancies/live births (↑)
	Fernando et al., 2018 [66]	Pilot double-blind dose finding, placebo-controlled randomized clinical trial	160 women involved (first cycle of IVF or ICSI): -40 women supplemented with placebo (controls), -41 women supplemented with 2 mg melatonin, -39 women supplemented with 4 mg melatonin, -40 women supplemented with 8 mg melatonin.	2, 4, 8 mg twice per day from day 2 of their cycle until the night before oocyte retrieval.	-No differences in clinical pregnancy rate or live birth rate between any of the four groups (→) -No differences between the groups in total oocyte number, number of MII oocytes, number of fertilized oocytes, or the number or quality of embryos between the groups (→)
**CoenzymeQ10**	Giannubilo et al., 2018 [67]	Observational	30 women involved: -15 women not supplemented with CoQ10 -15 woman supplemented with CoQ10	200 mg/day in two daily administrations, with main meals for 30 days.	-Significant improvement of follicular fluid oxidative status with CoQ10 supplementation (↑)
	Bentov et al., 2014 [68]	Double-blind placebo controlled randomized trial	24 women involved: -14 women not supplemented -10 women supplemented with CoQ10	600 mg of CoQ10 once a day with breakfast, orally, or identical placebo capsules for up to three cycles if pregnancy did not occur. All subjects took either CoQ10 or placebo for two months.	-The rate of aneuploidy was 46.5% in the CoQ10 group compared to 62.8% in the control (↑) -The clinical pregnancy rates comparable between CoQ10 group and the controls (→)
	Xu et al., 2018 [69]	Randomized controlled trial	169 women involved: -93 women not supplemented with CoQ10 -76 women supplemented with Coq10	200 mg three times a day, for a period of 60 days	-Increased number of retrieved oocytes, higher fertilization rate, and more high-quality embryos in women supplemented with CoQ10 (↑) -With CoQ10 intake, less women with cancelled embryo transfer and more women from treatment group with available cryopreserved embryos (↑) -Clinical pregnancy and live birth rates per embryo transfer and per one complete stimulation cycle higher in CoQ10 group but without statistical significance (↑)
**B-vitamins**	Gaskins et al., 2014 [70]	Prospective cohort	232 women involved	Diet was assessed before assisted reproductive technology treatment using a validated food frequency questionnaire.	-Higher folate intake was associated with higher rates of implantation, clinical pregnancy, and live birth (↑) -Live birth rates were 20% higher among women in the highest quartile of supplemental folate intake (more than 800 micrograms/day) than among women in the lowest quartile (less than 400 micrograms/day) (↑) -Higher supplemental folate intake was associated with higher fertilization rates and lower cycle failure rates before embryo transfer (↑)
	Gaskins et al., 2015 [71]	Prospective cohort	100 women (154 ART cycles) involved	Diet was assessed before assisted reproductive technology treatment using a validated food frequency questionnaire.	-Women in the highest quartile of serum folate (>26.3 ng/mL) had 1.62 times the probability of live birth compared with women in the lowest quartile (<16.6 ng/mL). Women in the highest quartile of serum vitamin B-12 (>701 pg/mL) had 2.04 times the probability of live birth compared with women in the lowest quartile (<439 pg/mL) (↑)
	Murto et al., 2014 [72]	Prospective case–control	368 women involved: -180 women with unexplained infertility -188 women in the control group	A questionnaire was used to assess general background and use of dietary supplements.	-Women with unexplained infertility used significantly more folic acid supplements and had better folate status than fertile women (↑) -No positive effect on pregnancy outcome after intake of folic acid supplements in unexplained infertility women (→)
	Murto et al., 2015 [73]	Prospective observational	340 women involved: -180 with unexplained infertility -86 with male-factor infertility -74 with female infertility	A questionnaire was used to assess general background data and use of dietary supplements	-Women in the infertility group used significantly more folic acid supplements and had better folate status than fertile women (↑) -no association between pregnancy outcome and folic acid intake, folate status or MTHFR gene variations (→)
	Haggarty et al., 2006 [74]	Prospective cohort	602 women involved	Folate and vitamin B12 assessed with a questionnaire	-No association between folate and vitamin B_12_ levels and likelihood of a successful pregnancy (→)
**Vitamin C**	Griesinger et al., 2002 [75]	Double-blind prospective randomized	619 women involved: -158 women supplemented with placebo (controls) -172 women supplemented with l-ascorbic acid (1 gr) -153 women supplemented with l-ascorbic acid (5 gr) -136 women supplemented with l-ascorbic acid (10 gr)	Four groups of supplementation: 1 gr, 5 gr, and 10 gr. Daily oral intake from the day of follicle aspiration and during the luteal phase (14 days).	-No differences in clinical pregnancy rate and implantation rate in statistical logistic regression analysis between the four intake groups (→)
	Lu et al., 2018 [76]	Randomized controlled trial	377 women involved: -132 women without endometriosis not supplemented -108 women with endometriosis not supplemented -137 patients with endometriosis supplemented	1000 mg/ day of oral Vitamin C from 2 months before IVF-ET treatment until 2 weeks after embryo transfer.	-Vitamin C levels in serum and follicular fluid significantly increased, while oxidative stress markers resulted unaffected (→) -No significant differences in the fertilization rate, implantation rate, or clinical pregnancy rate among the three study groups (→)
	Crha et al., 2003 [77]	Prospective	76 women involved: -38 smokers -38 non-smokers.Half the women (19 smokers and 19 non-smokers) were supplemented with vitamin C.	Daily doses of 500 mg during the period of hormonal stimulation.	-Impact of Vitamin C supplementation in terms of number of pregnancies, with a greater impact on the number of pregnancies in the non-smokers’ group (↑)

**Table 3:** Experimental in vitro studies about antioxidants in combination.

Authors	Models	Study Design	Antioxidants	Results
Truong et al., 2016 [78]	Mouse (C57BL6)	In vitro	10 µM acetyl-l-carnitine + 10 µM *N*-acetyl-l-cysteine +5 µM α-lipoic acid	-Antioxidants significantly increased mouse blastocyst cell numbers when all three were used in combination in 20% oxygen (↑)
Truong et al., 2017 [79]	Mouse (C57BL6)	In vitro	10 μM Acetyl-l-Carnitine, 10 μM *N*-Acetyl-l-Cysteine, 5 μM α-Lipoic Acid	-Increase in both blastocyst trophectoderm and inner cell mass cells numbers (↑) -No differences in embryo developmental rates and blastocyst cell number when antioxidants were present only in the sperm medium (→) -Faster time of cleavage with antioxidants supplementation (↑) -Levels of H_2_O_2_ significantly reduced in pronucleate oocytes cultured in the presence of antioxidants (↑)
Nikseresht et al., 2017 [80]	Mouse (NMRI)	In vitro	100 μM β-mercaptoethanol + 100 μM Cysteamine	-β-mercaptoethanol and Cysteamine significantly increased the rate of IVM and oocyte evolution, and embryo formation in culture medium (↑)
Caamaño et al., 1998 [81]	Bovine	In vitro	0.63 or 6.9 microM l-cysteine and 0, 10, or 100 microM beta-mercaptoethanol	-Blastocyst formation did not differ between 10 or 100 microM β-mercaptoethanol but was higher in both groups than for embryos cultured in the absence of β-mercaptoethanol (↑) -The rate of blastocyst formation increased for embryos cultured in the higher concentration of l-cysteine (↑) -Embryos cultured in the presence of β-mercaptoethanol, either 10 microM or 100 microM, had more cells than embryos cultured without β-mercaptoethanol (↑)

**Table 4:** Human clinical studies about antioxidants and multivitamins in combination during COS for IVF programs.

Authors	Study Design	Antioxidants and Supplementation	Patients Enrolled	Results
Fatemi et al., 2017 [82]	Randomized controlled trial	vitamin E (400 mg/day) + vitamin D3 (50,000 IU/one in two weeks) prior to combined oral contraceptive pills (COCP) intake and continued until hCG administration (approximately 8 weeks).	90 women involved: -46 women supplemented with placebo (controls) -44 women supplemented with vitamin D3 + vitamin E	-Clinical pregnancy and implantation rates significantly higher in the treatment group (↑) -Significant increase in serum malonialdehyde and significant decrease in serum total antioxidant capacity (TAC) after treatment (↑)
Ozkaya et al., 2010 [83]	Randomized controlled trial	Oral multivitamin and mineral tablet (Megadyn Pronatal Film Tablet, Mecome, Turkey). Daily intake, for 45 days before serum and follicular fluid collection.	69 women involved: -13 women supplemented with placebo -30 women constituted the IVF group supplemented with placebo -26 women received oral multivitamin and mineral tablets	-Multivitamin and mineral supplementation in serum and follicular fluid ameliorated the antioxidant status by decreasing oxidative stress (↑)
Youssef et al., 2014 [84]	Randomized controlled trial	Oral antioxidants medication containing: Vitamin A 3000 IU, vitamin E 15 IU, vitamin C 90 mg, zinc 11 mg, molybdenum 45 µg, selenium 55 µg, biotin 10 µg, and mixed bioflavonoid 100 mg (Octatron ^®^ Nerhadou International)	218 women involved: -106 women not supplemented -112 women received supplementation	-No significant changes between the groups as regards number of mature (MII) oocytes and clinical pregnancy rate per woman randomized (→)
